# Reproducibility of Single-Pulse, Paired-Pulse, and Intermittent Theta-Burst TMS Measures in Healthy Aging, Type-2 Diabetes, and Alzheimer’s Disease

**DOI:** 10.3389/fnagi.2017.00263

**Published:** 2017-08-21

**Authors:** Peter J. Fried, Ali Jannati, Paula Davila-Pérez, Alvaro Pascual-Leone

**Affiliations:** ^1^Berenson-Allen Center for Noninvasive Brain Stimulation, Division of Interventional Cognitive Neurology, Beth Israel Deaconess Medical Center, Harvard Medical School, Boston MA, United States; ^2^Departamento de Medicina, Facultade de Ciencias da Saúde, Universidade da Coruña A Coruña, Spain; ^3^Institut Guttman de Neurorehabilitació, Universitat Autónoma de Barcelona Barcelona, Spain

**Keywords:** reproducibility of results, transcranial magnetic stimulation, cortical plasticity, aging, Type-2 diabetes, Alzheimer’s disease

## Abstract

**Background:** Transcranial magnetic stimulation (TMS) can be used to assess neurophysiology and the mechanisms of cortical brain plasticity in humans *in vivo*. As the use of these measures in specific populations (e.g., Alzheimer’s disease; AD) increases, it is critical to understand their reproducibility (i.e., test–retest reliability) in the populations of interest.

**Objective:** Reproducibility of TMS measures was evaluated in older adults, including healthy, AD, and Type-2 diabetes mellitus (T2DM) groups.

**Methods:** Participants received two identical neurophysiological assessments within a year including motor thresholds, baseline motor evoked potentials (MEPs), short- and long-interval intracortical inhibition (SICI, LICI) and intracortical facilitation (ICF), and MEP changes following intermittent theta-burst stimulation (iTBS). Cronbach’s α coefficients were calculated to assess reproducibility. Multiple linear regression analyses were used to investigate factors related to intraindividual variability.

**Results:** Reproducibility was highest for motor thresholds, followed by baseline MEPs, SICI and LICI, and was lowest for ICF and iTBS aftereffects. The AD group tended to show higher reproducibility than T2DM or controls. Intraindividual variability of baseline MEPs was related to age and variability of RMT, while the intraindividual variability in post-iTBS measures was related to baseline MEP variability, intervisit duration, and Brain-derived neurotrophic factor (*BDNF*) polymorphism.

**Conclusion:** Increased reproducibility in AD may reflect pathophysiological declines in the efficacy of neuroplastic mechanisms. Reproducibility of iTBS aftereffects can be improved by keeping baseline MEPs consistent, controlling for *BDNF* genotype, and waiting at least a week between visits.

**Significance:** These findings provide the first direct assessment of reproducibility of TMS measures in older clinical populations. Reproducibility coefficients may be used to adjust effect- and sample size calculations for future studies.

## Introduction

Transcranial magnetic stimulation (TMS) is a non-invasive means of electrically stimulating the brain through electromagnetic induction. TMS can be applied as single-pulse TMS to assess cortical reactivity, paired-pulse TMS to probe intracortical inhibition and facilitation, and patterned trains of pulses, termed rTMS, to induce changes in cortical excitability and metabolism that last beyond the stimulation itself. When directed to the primary motor cortex, the aftereffects of rTMS are typically measured as a change in cortical reactivity, i.e., the average amplitude of MEPs elicited by single-pulse TMS. In most instances, continuous low-frequency (∼1 Hz) rTMS tends to reduce MEP amplitude, while on-off patterns of higher frequency (5–20 Hz) rTMS tend to increase MEP amplitude. These neuromodulatory effects of rTMS are thought to rely on mechanisms of brain plasticity related to LTD and LTP, respectively (Pascual-Leone et al., 1994; Hallett, 2007).

Over the past decade, an ultra high-frequency patterned rTMS application termed TBS has emerged as a potential means to generate greater and longer-lasting neuromodulatory effects with a shorter duration of stimulation (Huang et al., 2005). TBS consists of 50 Hz pulse-triplets repeated every 200 ms in one of two protocols that parallel low- and high-frequency rTMS: 40 s (600 pulses) of cTBS reduces MEP amplitude by about 10–25% for about 50 min, while the same number of pulses delivered in a 2-s on, 8-s off iTBS pattern for 190 s increases MEP amplitude by about 15–35% for 60 min (Wischnewski and Schutter, 2015). These TBS protocols have been used to identify age-related changes in the mechanisms of plasticity across the lifespan in healthy individuals (Freitas et al., 2011b) and reveal altered neuroplastic mechanisms in autism spectrum disorders (Oberman et al., 2012), traumatic brain injury (Tremblay et al., 2015), schizophrenia (McClintock et al., 2011), Type-2 diabetes (Fried et al., 2016), and AD (Koch et al., 2012).

The growth in popularity of TMS techniques has led to an increased focus on the sources of inter- and intraindividual variability. For example, it has been demonstrated that activation of the target muscle prior to, during, or immediately after TBS can influence its effects on motor cortex excitability (Huang et al., 2008; Iezzi et al., 2008; Goldsworthy et al., 2014). In addition, carriers of the *BDNF*-Met allele may show altered response to neuromodulation paradigms including TBS (Cheeran et al., 2008; Lee et al., 2013; Di Lazzaro et al., 2015). Despite increased attention, only four studies (Hinder et al., 2014; Vernet et al., 2014; Vallence et al., 2015; Schilberg et al., 2017) have directly assessed the reproducibility of TBS aftereffects, and these largely focused on young healthy individuals. Similarly, while there have been more studies investigating the reproducibility of single and paired-pulse TMS measures, only two (Kimiskidis et al., 2004; Fleming et al., 2012) included subjects over the age of 50, and only one (Christie et al., 2007) exclusively recruited individuals over 65 years. As interest grows in using TMS and TBS to assess the intracortical and corticospinal excitability and the efficacy of neuroplastic mechanisms in older clinical populations (Freitas et al., 2011b; Di Lorenzo et al., 2016; Fried et al., 2016), it is critical to understand the reliability of these techniques in the populations of interest. The present study aims to fill this void through a direct assessment of the reproducibility of iTBS aftereffects and other common single- and paired-pulse TMS-based neurophysiological measurements in older adults, including those with impairments in cognition or glucose metabolism. The results from this study will serve as a guidepost for understanding how biomarkers of cortical reactivity and plasticity change with age or are affected by common diseases such as Type-2 diabetes and AD.

## Materials and Methods

### Human Participants

The study was carried out in accordance with the Declaration of Helsinki and the recommendations of the Institutional Review Board at Beth Israel Deaconess Medical Center with written informed consent from all subjects.

Retrospective data was obtained from 36 adults (17 females) of mean age 62.9 years (range: 50–79 years), who had participated in research at the Berenson-Allen Center for Noninvasive Brain Stimulation at Beth Israel Deaconess Medical Center between May 2012 and May 2015. The participants were drawn from different populations: nine participants (four males, mean ± SD age: 67.7 ± 6.9 years) had a probable diagnosis of mild-to-moderate AD (McKhann et al., 2011) with a CDR = 1.0 and an MMSE score between 18 and 23; 15 participants (nine males, mean ± SD age: 63.4 ± 7.3 years) had Type-2 diabetes (T2DM) but were otherwise cognitively intact (MMSE ≥ 27), and the remaining 12 healthy controls (six males, mean ± SD age: 58.6 ± 9.1 years) were both cognitively intact (MMSE ≥ 27) and non-diabetic (hemoglobin A1c < 6.2%). AD participants consisted of individuals who were randomized to a Sham-control group for a proof-of-principle study on the combined impact of daily rTMS and cognitive training (Brem et al., manuscript submitted for publication). T2DM and control participants were recruited for a study on cortical plasticity in T2DM (Fried et al., 2016). None of the participants had any unstable medical condition or comorbidity. Saliva was obtained from 24 participants (10 controls, 10 T2DM, 4 AD) to assess *BDNF* and *APOE* polymorphisms. All participants underwent anatomical MRI scan, structured neurological exam, medical history review, formal neuropsychological testing, and two identical TMS visits. Median time between TMS visits was 14 days (range: 2–344 days). Average (±SD) start time for the two TMS visits was 10:57 (±1:07) for Visit-A and 10:37 (±0:55) for Visit-B. Blood glucose levels were assessed in all T2DM subjects at the beginning of each TMS visit for the purpose of establishing that glucose levels were within a normative range defined *a priori* as 80–200 mg/dL.

### Magnetic Resonance Imaging

A T1-weighted anatomical MRI scan was obtained in all participants and used for neuronavigation. Scans were completed on a 3T scanner (GE Healthcare, Ltd., United Kingdom) using a 3D spoiled gradient echo sequence: 162 axial-oriented slices for whole-brain coverage; 240-mm isotropic field-of-view; 0.937-mm × 0.937-mm × 1-mm native resolution; flip angle = 15°; TE/TR ≥ 2.9/6.9 ms; duration ≥ 432 s.

### Transcranial Magnetic Stimulation

All parameters used in the study conformed to current recommended guidelines for the safe application of TMS endorsed by the IFCN (Rossi et al., 2009; Rossini et al., 2015). A Navigated Brain Stimulation system (Nexstim Plc, Finland) was used to identify the hand region of the left primary motor cortex and ensure consistent targeting throughout each TMS visit. At the first visit, the hotspot for the FDI muscle was marked on the participant’s MRI. The hotspot was defined as the site from which single-pulse TMS elicited MEPs that were more consistent and higher in amplitude in FDI than in either the abductor pollicis brevis or abductor digiti minimi muscles. The hotspot was reassessed at the second visit using the first visit as a reference. Following IFCN guidelines (Rossini et al., 2015), RMT (using both mono- and biphasic pulses) and AMT (using biphasic pulses) were measured. We defined RMT as *the lowest intensity of stimulation that elicited MEPs* ≥*50* μV *in at least five of ten pulses in the relaxed FDI*, and AMT as *the lowest intensity that elicited MEPs* ≥*200* μV *in at least 5 of 10 pulses with the FDI slightly contracted*. Single and paired-pulse TMS trials were separated by a randomized 5000–6000 ms interval to avoid applying patterned repetitive stimulation.

#### Paired-Pulse TMS

Neuronavigated paired-pulse TMS was applied using a handheld monophasic (posterior–anterior in the brain) figure-of-eight focal coil (Nexstim Plc, Finland). Three protocols were utilized: SICI, consisting of a subthreshold (80% RMT) conditioning pulse followed 3 ms by a suprathreshold (120% RMT) test pulse; ICF (subthreshold conditioning pulse followed 12 ms by a suprathreshold pulse); and LICI (suprathreshold conditioning pulse followed 100 ms by a suprathreshold test pulse) (Valls-Solé et al., 1992; Kujirai et al., 1993). A block of single monophasic-TMS pulses at 120% RMT provided a measure of unconditioned cortico-motor reactivity. Each block consisted of 50 trials and individual MEP amplitudes > 2.5 SD from the mean were excluded. Conditioned MEPs from SICI, LICI, and ICF blocks were averaged and expressed as the percent change from the unconditioned block. Paired-pulse measures could not be performed in two participants in whom RMT exceeded 83% of maximum stimulator output.

#### Theta-Burst TMS

Neuronavigated iTBS was applied to participants using a handheld passive-cooling fluid-filled figure-of-eight coil (MCF-B65; 75 mm outer wing diameter) attached to a MagPro X100 stimulator (MagVenture A/S, Denmark). Intensity was 80% of AMT. The pattern was a 2-s train of biphasic bursts (three pulses at 50 Hz) repeated every 200 ms (30 pulses per train). Trains were repeated 20 times with an eight-second inter-train interval (600 pulses, 192 s). This protocol has been shown to potentiate cortico-motor reactivity for up to 60 min in healthy individuals (Huang et al., 2005; Wischnewski and Schutter, 2015).

Prior to iTBS, participants received three blocks of 30 single TMS pulses at 120% RMT using a hand-held biphasic (anterior–posterior, posterior–anterior in the brain) figure-of-eight coil (Nexstim Plc). Cortico-motor reactivity was reassessed in blocks of 30 TMS pulses at 5, 10, 20, 30, 40, and 50 min post-iTBS. The peak-to-peak amplitude of each recorded MEP was measured automatically. For each block, individual MEPs > 2.5 SD from the mean were excluded. All 90 pre-iTBS trials were averaged as a measure of baseline cortico-motor reactivity. MEP trials were averaged for each post-iTBS block and expressed as the percent change from baseline. MEPs were not obtained at 30-min post-iTBS in two participants and 50-min post-iTBS in one participant. In those participants, the corresponding time-point from the other visit was therefore excluded from subsequent analyses.

### Statistical Analyses

Neurophysiological data included three motor thresholds (mono- and biphasic RMT and biphasic AMT; expressed as percent of maximum stimulator output intensity), two measures of cortico-motor reactivity (unconditioned MEPs elicited with the monophasic coil that was used to assess the effects of the paired-pulse paradigms and baseline MEPs elicited with the biphasic coil that were used to assess the impact of iTBS), three paired-pulse measures (SICI, LICI, ICF; with the average amplitude of the conditioned MEPs expressed as the percent change the amplitude of unconditioned MEPs), and the six post-iTBS time-points (Post05, Post10, Post20, Post30, Post40, and Post50; with the average amplitude of MEPs from each time-point expressed as the percent change from the pre-iTBS baseline average). From the iTBS modulation, three further measures of plasticity were calculated: the Max+, or greatest change in MEP amplitude across all six time-points; the summed area under-the-curve for the first 20-min post-iTBS (AUC_0-20_), corresponding to the period of peak effect in neurotypical individuals (Wischnewski and Schutter, 2015); and the summed area under-the-curve across all post-iTBS time-points (AUC_0-50_). The area under-the-curve was calculated as the summed products of the average %Δ in MEP amplitude at two consecutive time-points and the time in minutes between them.

For all neurophysiological measures, Cronbach’s α coefficients (Cronbach and Warrington, 1951) were calculated between the two visits to assess test–retest reliability. The α coefficient provides a measure of internal consistency of a set of items (Tavakol and Dennick, 2011), in this case, the same subjects tested on two separate occasions. The α coefficient was calculated for all subjects together and for each group (AD, T2DM, controls) separately using the free online software program *Cronbach alpha* (v1.0.3^1^) (Wessa, 2016).

Reliability coefficients, such as the α, can be used to adjust effect sizes (Baugh, 2002; Wright, 2014). Using this approach, it is possible to predict how the reproducibility of a given measure might affect a hypothetical effect size, which in turn could be used in a sample size calculation that takes into consideration the reproducibility (or lack thereof) of the measure of interest. To illustrate this point and provide a resource for future studies, adjustments for each measure were made to a hypothetical Cohen’s *d* effect size of 0.5, which corresponds to a within-subject change of half a standard deviation, and is considered a medium effect size (Cohen, 1992). First, a hypothetical, or *idealized*, Cohen’s *d* is converted into an *r* (Cohen, 1988, p. 23):

(1) $$\begin{array}{c} r_{\mathrm{IDEALIZED}}\;=\;d{}_{\mathrm{IDEALIZED}}/{\left(d^{2}{}_{\mathrm{IDEALIZED}}\;+\;4\right)}^{0.5} \end{array}$$

This *idealized r* is then adjusted for unreliability using the Cronbach’s α (Wright, 2014):

(2) $$\begin{array}{c} r^{2}{}_{\mathrm{ADJUSTED}}\;=\;r^{2}{}_{\mathrm{IDEALIZED}}*\;\alpha^{0.5} \end{array}$$

Finally, the corrected *r* is converted back into an adjusted *d* (Friedman, 1968, p. 246):

(3) $$\begin{array}{c} d_{\mathrm{ADJUSTED}}\;=\;(2*r_{\mathrm{ADJUSTED}})/{\left(1\;-\;r^{2}{}_{\mathrm{ADJUSTED}}\right)}^{0.5} \end{array}$$

To investigate factors associated with intraindividual variability, additional analyses were performed in JMP Pro (v12.1.0^2^) using a normal distribution and a two-tailed 95% confidence interval. Given the exploratory nature of these analyses, individual *p*-values were not adjusted for multiple comparisons and should be interpreted accordingly. For between-groups comparisons, the sample sizes in the present study provided 0.80 power to detect a medium effect size (Cohen’s *d* = 0.54). The first set of analyses concerned correlations between variables that were collected at each visit and thus were performed using the net difference between Visits A and B (Δ_B-A_) so that the direction of change between visits was taken into account. These analyses included: (1) how differences in baseline MEP amplitude relate to differences in RMT (for both mono- and biphasic pulses); (2) how differences in SICI, LICI, and ICF relate to differences in unconditioned monophasic MEP amplitudes and RMT; and (3) how differences in post-iTBS measures relate to differences in baseline biphasic MEP amplitudes, RMT, and AMT. The second set of analyses concerned factors, such as group, gender, age, inter-visit interval, and *BDNF* and *APOE* polymorphisms, that were assessed only once per participant. Multiple linear regression analyses were performed on the absolute value of the inter-visit difference (|Δ_B-A_|) to account for the amount of change between visits in either direction.

## Results

Data on motor thresholds, baseline cortico-motor reactivity measures, changes in MEP amplitude from the paired-pulse TMS and post-iTBS plasticity measures are shown in **Table 1**.

**Table 1:** Neurophysiological Measures.

	Visit-A	Visit-B	Δ_B-A_	|Δ_B-A_|
	Mean ±*SD*	Mean ±*SD*	Mean ±*SD*	Mean ±*SD*
**Motor threshold (% MSO)**				
RMT monophasic	63.08 ± 13.4	65.00 ± 13.8	1.92 ± 4.6	3.86 ± 3.1
RMT biphasic	45.03 ± 11.7	44.72 ± 10.5	–0.31 ± 5.4	3.81 ± 3.8
AMT biphasic	43.97 ± 11.6	43.83 ± 10.0	–0.20 ± 6.6	4.26 ± 5.1
**Baseline MEPs (mV)**				
Monophasic	0.91 ± 0.9	0.89 ± 1.0	0.00 ± 0.8	0.48 ± 0.6
Biphasic	1.35 ± 1.0	1.30 ± 1.1	–0.05 ± 1.1	0.74 ± 0.8
**Paired-pulse (%Δ)^†^**				
SICI	–23.06 ± 65.2	–12.36 ± 59.0	10.70 ± 59.0	41.42 ± 42.8
LICI	–49.73 ± 77.6	–67.21 ± 47.6	–17.48 ± 74.4	34.64 ± 68.0
ICF	118.64 ± 203.0	145.36 ± 341.6	26.73 ± 368.4	166.55 ± 328.4
**Post-iTBS (%Δ)^††^**				
5 min post-iTBS	13.56 ± 51.3	27.50 ± 71.3	13.94 ± 75.5	53.61 ± 54.2
10 min post-iTBS	11.81 ± 46.4	10.97 ± 51.9	–0.83 ± 66.1	47.56 ± 45.3
20 min post-iTBS	–10.11 ± 43.1	18.44 ± 62.5	28.56 ± 64.0	51.33 ± 47.1
30 min post-iTBS	–0.41 ± 43.2	6.78 ± 60.2	7.15 ± 66.2	48.26 ± 45.0
40 min post-iTBS	7.94 ± 53.6	20.61 ± 98.0	12.67 ± 92.9	63.11 ± 68.5
50 min post-iTBS	–3.31 ± 49.0	12.36 ± 80.3	16.37 ± 90.5	58.66 ± 70.2
Maximum facilitation	52.22 ± 52.5	85.31 ± 98.1	33.08 ± 93.6	62.53 ± 76.6
**Area under-the-curve (%Δ^∗^time)^††^**				
0–20 min post-iTBS	105.28 ± 690.4	313.06 ± 901.9	206.08 ± 948.1	711.64 ± 649.4
0–50 min post-iTBS	47.71 ± 1609.9	738.89 ± 2529.1	737.57 ± 2422.6	1759.91 ± 1800.2

*Δ_*B*–*A*_, net inter-visit difference; |Δ_*B*–*A*_|, absolute inter-visit difference; MSO, maximum stimulator output; RMT, resting motor threshold; AMT, active motor threshold; MEPs, motor evoked potentials; SICI, short-interval intracortical inhibition; LICI, long-interval intracortical inhibition; ICF, intracortical facilitation. ^†^Percent change calculated from monophasic baseline; ^††^percent change calculated from biphasic baseline.*

### Reproducibility of Neurophysiological Measures

**FIGURE 1:**
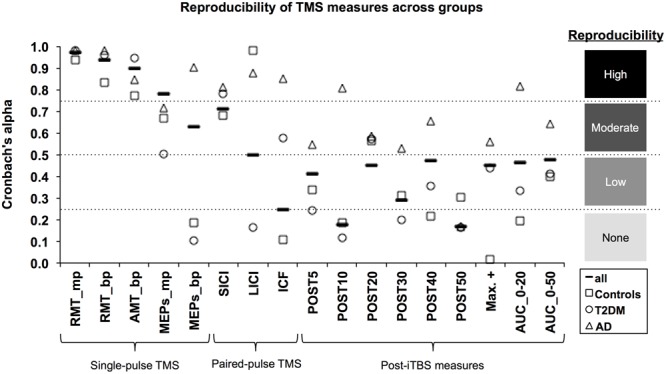
Reproducibility of TMS measures across groups. The Cronbach’s α coefficient (*y*-axis) was calculated as an index of reliability for each TMS-based measure (*x*-axis). α coefficients were calculated for all subjects (solid line marker) as well as for each group: Alzheimer’s disease (AD; triangle marker); Type-2 diabetes mellitus (T2DM; circle marker); and non-AD/non-T2DM controls (square marker). Following the approach of Portney and Watkins (2009), reproducibility was categorized as high (α ≥ 0.75), moderate (0.50 ≤ α < 0.75), low (0.25 ≤ α < 0.50), and very low to none (α < 0.25). mp, monophasic; bp, biphasic; RMT, resting motor threshold; AMT, active motor threshold; MEPs, motor evoked potentials; SICI, short-interval intracortical inhibition; LICI, long-interval intracortical inhibition; ICF, intracortical facilitation; iTBS, intermittent theta-burst stimulation; POST, minutes post-iTBS; Max+, maximum facilitation; AUC, area under-the-curve.

**Figure 1** shows coefficients for all measures and all groups. Hypothetical effect- and sample sizes adjusted for these α coefficients are shown in **Table 2**. Following criteria for categorizing reproducibility in neurophysiological assessments (Portney and Watkins, 2009), we defined α ≥ 0.75 as high reproducibility, 0.50 ≤ α < 0.75 as moderate reproducibility, 0.25 ≤ α < 0.50 as low reproducibility, and α < 0.25 as very low to no reproducibility.

**Table 2:** Alpha coefficients and corresponding adjusted effect and sample sizes.

					Reproducibility-adjusted				Additional *n* required			
	Cronbach’s α coefficients				Cohen’s *d* (0.50)				(power = 0.80)			
	All	HC	T2DM	AD	All	HC	T2DM	AD	All	HC	T2DM	AD
**Motor thresholds**												
RMT monophasic	0.97	0.94	0.98	0.98	0.50	0.49	0.50	0.50	0	1	0	0
RMT biphasic	0.94	0.83	0.96	0.98	0.49	0.48	0.49	0.50	1	3	1	0
AMT biphasic	0.90	0.77	0.95	0.85	0.49	0.47	0.49	0.48	2	4	1	3
**Baseline MEPs**												
Monophasic	0.78	0.67	0.50	0.72	0.47	0.45	0.42	0.46	4	7	14	6
Biphasic	0.63	0.19	0.11	0.90	0.44	0.32	0.28	0.49	9	44	69	2
**Paired-pulse measures**												
SICI	0.71	0.68	0.78	0.81	0.46	0.45	0.47	0.47	6	7	4	4
LICI	0.50	0.98	0.17	0.88	0.42	0.50	0.31	0.48	14	0	49	2
ICF	0.25	0.11	0.58	0.85	0.35	0.28	0.43	0.48	34	69	10	3
**Post-iTBS measures**												
5 min post-iTBS	0.41	0.34	0.24	0.55	0.40	0.38	0.34	0.43	19	24	34	12
10 min post-iTBS	0.18	0.19	0.12	0.81	0.32	0.32	0.29	0.47	46	44	64	4
20 min post-iTBS	0.45	0.56	0.57	0.59	0.41	0.43	0.43	0.43	16	11	11	10
30 min post-iTBS	0.29	0.31	0.20	0.53	0.36	0.37	0.33	0.42	28	26	41	12
40 min post-iTBS	0.47	0.22	0.35	0.65	0.41	0.34	0.38	0.45	15	38	23	8
50 min post-iTBS	0.17	0.30	0.16	0.17	0.31	0.37	0.31	0.31	48	27	49	11
Maximum facilitation	0.45	0.02	0.44	0.56	0.41	0.17	0.40	0.43	16	234	17	11
**Area under-the-curve**												
0–20 min post-iTBS	0.46	0.20	0.33	0.82	0.41	0.33	0.37	0.47	15	42	24	3
0–50 min post-iTBS	0.48	0.40	0.41	0.64	0.41	0.39	0.40	0.44	15	19	19	8

*HC, healthy controls; T2DM, Type-2 diabetes mellitus; AD, Alzheimer’s disease; RMT, resting motor threshold; AMT, active motor threshold; MEPs, motor evoked potentials; SICI, short-interval intracortical inhibition; LICI, long-interval intracortical inhibition; ICF, intracortical facilitation.*

Considering all groups combined, the three motor thresholds had high reproducibility (α’s > 0.90). For baseline MEPs elicited at 120% of RMT, monophasic (α = 0.78) and biphasic pulses (α = 0.62) showed high and moderate reproducibility, respectively. Among the paired-pulse measures, reproducibility was moderate for SICI (α = 0.71) and LICI (α = 0.54), while ICF had very low to no reproducibility (α = 0.25). All post-iTBS measures demonstrated low reproducibility (α’s = 0.29–0.48); except for Post10 (α = 0.18) and Post50 (α = 0.17), which were not reproducible.

Considering each group separately, α coefficients tended to be higher for the AD group than for T2DM and controls. In particular, the AD group demonstrated high reproducibility for biphasic MEPs (α = 0.90), all three paired-pulse protocols (α’s > 0.81), Post10 (α = 0.81), and AUC_0-20_ (α = 0.82). Further, reproducibility in AD was moderate (α’s = 0.53–0.65) for all remaining post-iTBS measures, except Post50, which was not reproducible (α = 0.16). By comparison, both controls and T2DM individuals showed moderate reproducibility (α’s = 0.50–0.67) for baseline monophasic MEPs and no reproducibility (α’s = 0.11–0.19) for baseline biphasic MEPs. One positive outlier was LICI, which showed high reproducibility in controls (α = 0.98).

### Relationships between the Net Differences of Neurophysiological Measures

For measures assessed with a monophasic pulse, there was no significant relationship between the Δ_B-A_ of baseline MEP amplitudes and the Δ_B-A_ of RMT, *R*_31_ = 0.01, *p* = 0.934. Similarly, there were no significant relationships between the Δ_B-A_ of any of the paired-pulses measurements with the Δ_B-A_ of either RMT or baseline MEP amplitudes (|*R*|’s < 0.27, *p*’s > 0.13).

**FIGURE 2:**
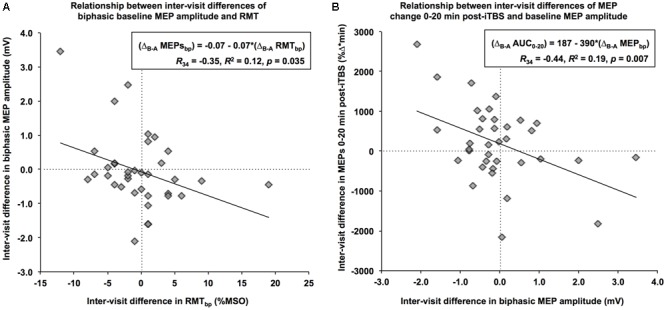
Relationships between the net differences of neurophysiological measures. **(A)** Using a biphasic pulse, an increase in RMT of 1% of maximum stimulator output from Visit-A to Visit-B (*x*-axis) was associated with a decrease of 0.07 mV in baseline MEP over the same period (*y*-axis). **(B)** An increase of 1-mV in baseline MEP amplitude from Visit-A to Visit-B (*x*-axis) was associated with an inter-visit decrease of 390 %Δ^∗^min in the MEP change during the first 20 min (*y*-axis).

When using a biphasic coil, the Δ_B-A_ of baseline MEP amplitudes was significantly correlated with the Δ_B-A_ of RMT, *R*_34_ = -0.35, *p* = 0.035. Specifically, a 1% (maximum stimulator output) increase in the net difference of RMT was associated with a 70-μV decrease in the net difference of baseline MEP amplitude (**Figure 2A**). Further, the Δ_B-A_’s for all iTBS plasticity measures except Post30 and Post40 were significantly correlated with the Δ_B-A_ of biphasic baseline MEPs amplitudes (*R*’s<-0.36, *p* < 0.04). In all cases, an increase in the net difference of baseline MEP amplitudes was associated with a decrease in the inter-visit difference of post-iTBS facilitation. This relationship was most apparent for AUC_0-20_, where a 1-mV increase in the inter-visit difference of baseline MEP amplitude was associated with a 390 (%Δ^∗^min) decrease in the net difference of the AUC (**Figure 2B**). By contrast, there were no significant relationships between the Δ_B-A_ of any of the iTBS plasticity measures with the Δ_B-A_ of either RMT or AMT (|*R*|’s < 0.32, *p*’s > 0.07). These results indicate that as much as 23% of the visit-to-visit variability in iTBS plasticity measures can be accounted for by the variability in the baseline MEP amplitude, which in turn is impacted by the variability in RMT.

In the T2DM subjects, blood glucose levels did not differ significantly between visits (*p* > 0.1) and no significant relationships were observed between changes in blood glucose levels and changes in any TMS measure between visits (*p*’s > 0.2).

### Analyses of the Absolute Difference between Visits

Controlling for the interval between visits, as well as the age and gender of participants, the linear models yielded no difference between groups in the |Δ_B-A_| of any neurophysiological measure (*F*’s < 2.2, *p*’s > 0.13). These results indicate that the absolute amount of change between visits in motor thresholds as well as baseline reactivity, paired-pulse, and plasticity measures were equivalent across AD, T2DM and control participants at the 0.05 level.

The multiple regression analyses indicated a significant relationship between the |Δ_B-A_| of monophasic MEPs and *age*, controlling for *group, gender*, and *inter-visit interval* (*F*_1,1_ = 5.62, *p* = 0.025). Specifically, a 1-year increase in participant age was associated with a 0.03 mV increase in the absolute visit-to-visit difference in the amplitude of biphasic MEPs. Similarly, there was a significant relationship between the |Δ_B-A_| of Post05 facilitation and *inter-visit interval*, controlling for *group, gender*, and *age* (*F*_1,1_ = 6.42, *p* = 0.017). Specifically, a 1-day increase in the interval between visits was associated with a 0.03 mV decrease in the absolute inter-visit difference in the %Δ in MEP amplitudes at Post05. None of the other relationships were significant (*F*’s < 4.0, *p*’s > 0.05).

**FIGURE 3:**
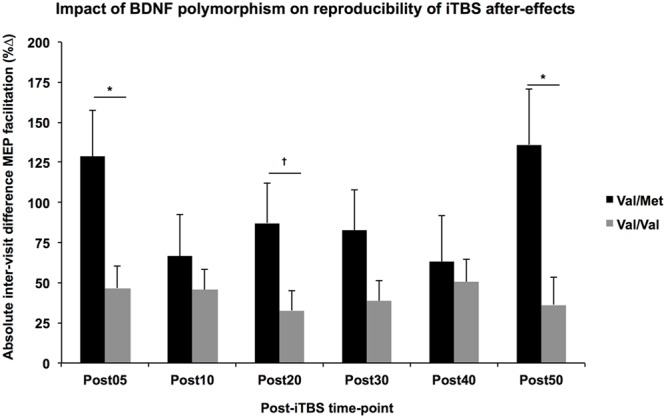
Impact of *BDNF* polymorphism on reproducibility of iTBS aftereffects. Controlling for Group, the absolute difference in MEP facilitation (*y*-axis) tended to be higher across all post-iTBS time-points (*x*-axis) in Val-Met carriers (black) than Val/Val homozygotes (gray). These differences were significant at 5- and 50-min post-iTBS, ^∗^*p* < 0.05, ^†^*p* < 0.1.

The multiple regression analyses demonstrated a significant effect of *BDNF* polymorphism on the |Δ_B-A_| of several post-iTBS measures after controlling for *Group* (**Figure 3**). Specifically, intraindividual variability was higher for *BDNF*-Met carriers than *BDNF*-Val homozygotes for Post05 (*F*_1,1_ = 6.76, *p* = 0.017) and Post50 (*F*_1,1_ = 6.79, *p* = 0.017), and AUC_0-20_ (*F*_1,1_ = 4.99, *p* = 0.037). By comparison, there was no significant effect of *APOE* status on the |Δ_B-A_| of any of TMS measures (*F*’s < 2.8, *p*’s > 0.1).

## Discussion

The potential of TMS-based assessments to provide meaningful insights into human neurophysiology is constrained by its variability. In particular, the intraindividual variability of a measure reduces its sensitivity to detect meaningful changes over time or in responses to an intervention. As TMS is increasingly applied in different neuropsychiatric conditions, it is crucial to evaluate its reproducibility in the target populations. The current study offers the first direct analysis of reproducibility of single- and paired-pulse TMS, and patterned repetitive TMS in older healthy adults and those with impairments in cognition or glucose metabolism. The results show that reproducibility varies considerably across measures and populations. Motor thresholds remain the gold standard in test–retest reliability; SICI and LICI tended to be more reproducible than ICF, though variability in LICI and ICF differed considerably across groups. Lastly, measures of LTP-like plasticity from iTBS were among the least reproducible, especially for older healthy and diabetic individuals.

Two recent studies in younger healthy individuals have reported higher intraindividual variability in the response to iTBS (Hinder et al., 2014; Schilberg et al., 2017). The present results, based on data from healthy older adults and those with impairments in either cognition or glucose metabolism, are more-or-less consistent with those reports in younger adults and suggest that variability in the aftereffects of iTBS remains a significant challenge to its use as a biomarker for the efficacy of neuroplastic mechanisms across the lifespan. Previous studies have identified factors such as prior exercise (McDonnell et al., 2013), ongoing voluntary activity (Huang et al., 2008), and other state-dependent effects (Silvanto et al., 2007), that can influence the efficacy of TBS and thus increase intraindividual variability (for a review, see Ridding and Ziemann, 2010). Other factors such as *BDNF* polymorphisms and baseline cortico-motor reactivity are discussed below. Some factors could be disease-specific, such as fluctuations in blood glucose levels in T2DM, though, importantly, glucose levels (within the range of 80–200 mg/dL, specified *a priori*) were not found to influence variability in the present study. Interestingly, the AD group showed numerically higher reproducibility coefficients for nearly all measures, including RMT, SICI and iTBS aftereffects, which several studies in AD have shown to be abnormal and/or predictive of disease severity or response to treatment (Liepert et al., 2001; Di Lazzaro et al., 2004; Koch et al., 2012, 2015; Brem et al., 2013; Balla et al., 2014). It is possible, however unlikely, that some aspect of the Sham treatment (e.g., daily study visits or interaction with study staff) that the AD group underwent had some stabilizing effect on their neurophysiology. This possibility could be investigated further by conducting test–retest assessments in a similar AD cohort over a similar timeframe that did not include significant changes to their regular schedule. A more likely explanation is that the same pathological processes that cause certain measures to be abnormal in AD also exert a stabilizing effect on TMS measures. In particular, the reduction in LTP-like plasticity following iTBS (and in some cases conversion to a LTD-like response) (Koch et al., 2011, 2012; Di Lorenzo et al., 2016) could reflect pathological changes in the brains of AD patients that reduce state-dependent effect. In any case, the relatively high reproducibility of most TMS measures in AD appears to validate their use as surrogate biomarkers of AD cortical pathology (Freitas et al., 2011a).

Reliability coefficients such as Cronbach’s α can be used to adjust effect sizes to account for the fact that those calculations are made under the implicit assumption of perfect test–retest reliability (Baugh, 2002; Wright, 2014). In other words, detecting a significant change of any given size in a longitudinal design is more difficult for an unreliable measure than for a reliable one. In turn, an adjusted effect size can be used to provide a more realistic estimate of the sample size required to observe the desired effect given the reproducibility of the measure being tested. **Table 2** shows adjustments to a hypothetical Cohen’s *d* of 0.5, which corresponds to a within-subject change of half a standard deviation, for each of the measures in the current analysis. **Table 2** also shows the adjustments in the sample sizes required to detect the attenuated effects. The results of the present analysis can thus be used to more accurately plan for future studies using TMS-based neurophysiological measures as prognostic biomarkers in older healthy, diabetic, and AD populations.

### Variability in Baseline MEP and Its Role in Post-iTBS Variability

Epidural recordings of cortico-spinal volleys in conscious humans receiving TMS have shown that depending on its shape, current direction, and intensity, a TMS pulse can result in direct depolarization of the layer-V pyramidal cell (D-waves) and/or indirect depolarization through local circuits of interneurons (I-waves) (Burke et al., 1993; Sakai et al., 1997). The use of posterior–anterior monophasic pulse waveforms, which primarily elicit early I-waves, yields higher reproducibility in measures of cortico-motor reactivity over biphasic stimulation, which elicits a more complex pattern of D-waves, and early and late I-waves depending on the intensity of stimulation (Di Lazzaro et al., 2003). Future studies should directly explore how the effect size and reproducibility of single-pulse, paired-pulse, and TBS-based TMS measures are influenced by physical TMS parameters such as pulse shape and duration, and induced current direction relative to the motor cortex.

The reproducibility of biphasic MEP amplitude (α = 0.62) was noticeably lower than that of biphasic RMT (α = 0.93). Given that MEPs were assessed using 120% of RMT, there appear to be factors that do not impact RMT but do add variability to batches of MEPs elicited at suprathreshold intensities. Moreover, the inter-visit change in biphasic baseline MEP amplitudes was inversely related to that of biphasic RMT, suggesting that stimulus-response curve (i.e., the relationship between TMS intensity and MEP size) itself varies across visits. It has been speculated that, at threshold intensities, the second half of the biphasic pulse (posterior–anterior in the present study) contributes primarily to the MEP, while at suprathreshold intensities, there is increasing influence of the first half of the pulse (anterior-posterior in the present study) (Di Lazzaro et al., 2003; Barker, 2017). There is at least some theoretical evidence that biphasic TMS pulses might be less effective than monophasic (posterior–anterior) at probing the neuromodulatory effects of TBS (Di Lazzaro and Rothwell, 2014). However, to our knowledge, this has never been directly investigated. Regardless, the relatively low reproducibility of baseline MEP amplitudes is an area of concern given that it is the basis on which post-iTBS measures are derived. Furthermore, a significant portion of the inter-visit variance in post-iTBS measures is accounted for by visit-to-visit difference in baseline MEP amplitudes. These results are consistent with a recent study showing variability in MEPs within a session is predictive of the response to cTBS (Hordacre et al., 2017). Furthermore, the present results imply that improving the consistency of baseline measures (within and across sessions) would decrease the variability of post-iTBS measures as well. Given that changes in stimulus-response curves might contribute to the changing relationship between RMT and suprathreshold MEP amplitudes, future studies should explore whether the reproducibility of these measures could be improved by choosing stimulation intensities based on individual stimulus-response curves rather than a fixed percent of RMT.

The use of neuronavigation has been shown to increase the consistency of MEPs (Julkunen et al., 2009). However, even with neuronavigation, handheld TMS remains prone to slight deviations in the position, orientation, and inclination of the TMS coil. Robot arms, such as the TMS Robot (Axilum^®^ Robotics, Strasbourg) have been shown to improve the consistency of trial-to-trial MEPs over handheld TMS (Foucher et al., 2012; Ginhoux et al., 2013). Typically, MEP trials are elicited with individually spaced TMS pulses at a specific frequency range (e.g., 5000–6000 ms in the present study) with some random jitter incorporated to reduce the likelihood of train effects. Several recent studies combining TMS with concurrent EEG have highlighted to role of pre-stimulus oscillatory activity on cortico-motor excitability. Mäki and Ilmoniemi (2010) demonstrated that MEP amplitudes are inversely correlated with the amplitudes of pre-stimulus midrange-beta oscillations (15–18 Hz) over the stimulated motor cortex. Similarly, Iscan et al. (2016) showed that the variability of pre-stimulus power in the upper alpha band (10–12 Hz) was predictive of variability in ICF trials. Alternatively, Berger et al. (2014) suggest that the instantaneous phase of EEG oscillations across a range of frequencies is more predictive of MEP amplitude than spectral power. Together, these studies suggest that technological advances that allow for closed-loop systems to trigger TMS pulses timed to real-time EEG rhythms should result in more consistent MEPs. While these approaches offer the potential to improve the trial-to-trial consistency of MEPs, whether they would translate to greater reproducibility across visits remains to be explored.

### Impact of Age and Time between Visits

**FIGURE 4:**
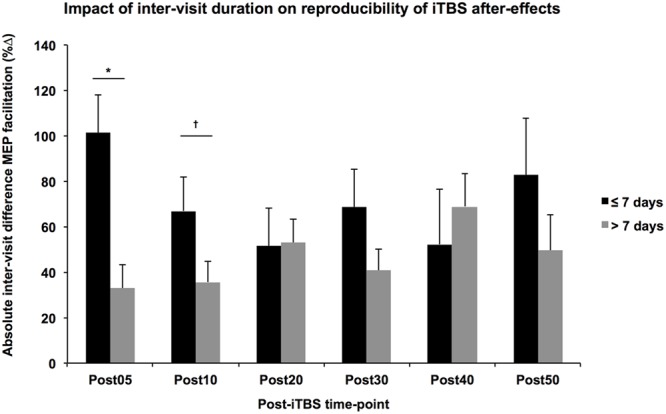
Impact of inter-visit duration on reproducibility of iTBS aftereffects. Controlling for Group, Age, and Gender, the absolute difference in MEP facilitation (*y*-axis) tended to be higher across all post-iTBS time-points (*x*-axis) in subjects that received their second visit within 7 days (black) than those whose second visit occurred greater than 7 days after the first (gray). These differences were significant at 5-min post-iTBS, ^∗^*p* < 0.05, ^†^*p* < 0.1.

While the multiple regression analyses did not show any significant difference between groups in terms of the absolute difference of the measures, participant age was significantly related to the absolute difference of monophasic baseline MEP amplitudes and *inter-visit duration* was significantly related to the absolute difference in Post05 facilitation, controlling for other factors such as *group* and *gender*. These results must be interpreted cautiously given the potential for Type-2 error in the present analysis. That the variability in baseline MEPs increases with age is not particularly surprising; however, its influence is not easily controlled, especially if the focus of the study is aging. It is more surprising that immediate iTBS aftereffects would be more consistent with greater time between visits. One possibility is that visits repeated under shorter intervals might be influenced by the iTBS from the previous visit, a phenomenon known as *metaplasticity*. It has been shown that the neuromodulatory effects of rTMS increase with consecutive daily application (Maeda et al., 2000; Valero-Cabré et al., 2008). Moreover, these metaplastic effects and state-dependent interactions may be altered by age (Opie et al., 2017) or neuropsychiatric disorders such as autism and Fragile X syndrome (Oberman et al., 2016). While the impact of multiple sessions separated by more than 24 h has not been well explored, a single application of iTBS was shown to alter the expression of GABA-precursor enzyme GAD67 for up to 7 days in the neocortex of rats (Trippe et al., 2009), suggesting the window for metaplastic effects might be longer than previously understood. Indeed, a follow-up analysis of the current data found that the absolute difference of Post05 facilitation was higher between visits conducted within 7 days than those separated by more than a week (**Figure 4**).

### Influence of *BDNF* Polymorphisms

The multiple regression analyses showed that the absolute difference of several post-iTBS measures was higher in subjects with a *BDNF*-Met allele. While the generalizability of these findings is limited by the small sample size, they nonetheless provide insight into the debate over the role of *BDNF* polymorphisms in shaping the effects of neuromodulation. Several studies have reported a reduced impact of repetitive TMS in Met carriers (Cheeran et al., 2008; Cirillo et al., 2012; Lee et al., 2013; Chang et al., 2014; Di Lazzaro et al., 2015), still others have reported no difference (Li Voti et al., 2011; Mastroeni et al., 2013). The current results suggest that this divergence in the literature may be due to the fact that the *BDNF* Met allele leads to more variability in the response to neuromodulation rather than simply blunting its effects.

### Additional Considerations

The present study was a retrospective analysis of data collected under different study protocols. As such there are a number of limitations, principally, the generally small and unequal sample sizes. While the Cronbach’s α is fairly robust to variations in sample size, the linear regression analyses are susceptible to limitations of sample size. As such, the analyses comparing absolute difference of visits between groups are likely underpowered to detect differences of the magnitude observed in the present study. Future studies with larger samples are needed to confirm the present findings. In addition, a number of the non-diabetic controls had hemoglobin A1c values indicating possible pre-diabetes, which may have contributed to the decreased reproducibility seen in this cohort. Further, HbA1c values were not available from the AD group, so the influence of impaired glucose metabolism could not be investigated in AD subjects, despite reports of high co-morbidity between AD and T2DM (Hölscher, 2011).

## Conclusion

Motor thresholds remain the gold standard for reproducibility of any TMS measure as demonstrated by high Cronbach’s α coefficients. Post-iTBS measures of LTP-like plasticity demonstrate low reproducibility by comparison. Reproducibility was higher in the AD group, possibly reflecting pathological rigidity of neurophysiological systems. A number of factors may contribute to the intraindividual variability of iTBS aftereffects, including *BDNF* polymorphisms and variability in baseline MEP amplitudes, from which post-iTBS measures are calculated. Future studies can use the α coefficients to adjust expected effect size and required sample size calculations.

Based on these conclusions, we offer the following recommendations for future studies to potentially reduce the intraindividual variability in TMS measures, especially in the iTBS-induced modulation of cortico-motor reactivity. We note that these recommendations are based on exploratory analyses performed in a relatively small and heterogeneous group of subjects and further confirmatory studies are needed. (1) Waiting at least 7 days between repeated visits can reduce the probability of metaplastic effects, at least in healthy individuals. (2) Whenever possible, *BDNF* polymorphism should be taken into account, either by adding *BDNF* Met carrier status as a covariate, or by splitting the data into subgroups. (3) To reduce intraindividual variability in baseline MEP amplitudes and any resulting impact of this variability on post-iTBS measures, we recommend considering the use of a stimulation intensity derived from individual stimulus-response curves, rather than using a fixed percent of RMT.

## Author Contributions

Study concept and design: PF and AP-L. Data collection: PF. Data analysis: PF and AJ. Data interpretation: PF, AJ, PD-P, and AP-L. Drafting manuscript: PF. Revising manuscript: PF, AJ, PD-P, and AP-L. All authors approved the final version and agree to be accountable for the content of the work.

## Conflict of Interest Statement

AP-L serves on the scientific advisory boards for Magstim, Nexstim, Neuronix, Starlab Neuroscience, Neuroelectrics, Axilum Robotics, Constant Therapy, and Neosync; and is listed as an inventor on several issued and pending patents on the real-time integration of transcranial magnetic stimulation with electroencephalography and magnetic resonance imaging. The other authors declare that the research was conducted in the absence of any commercial or financial relationships that could be construed as a potential conflict of interest.

## Footnotes

1 http://www.wessa.net/

2 http://www.jmp.com

## Abbreviations

AD: Alzheimer’s disease
AMT: active motor threshold
APOE: apolipoprotein-E
AUC_0-20_: area under-the-curve for 0–20 min post-iTBS
AUC_0-50_: area under-the-curve for 0–50 min post-iTBS
BDNF: brain-derived neurotrophic factor
CDR: clinical dementia rating
cTBS: continuous theta-burst stimulation
EEG: electroencephalography
FDI: first dorsal interosseous
ICF: intracortical facilitation
IFCN: International Federation of Clinical Neurophysiology
iTBS: intermittent theta-burst stimulation
LICI: long-interval intracortical inhibition
LTD: long-term depression
LTP: long-term potentiation
Max+: maximum facilitation
MEP: motor evoked potential
MMSE: mini-mental status exam
MRI: magnetic resonance imaging
Post05 … Post50: 5-min … 50-min post-iTBS
RMT: resting motor threshold
rTMS: repetitive transcranial magnetic stimulation
SICI: short-interval intracortical inhibition
T2DM: type-2 diabetes mellitus
TBS: theta-burst stimulation
TMS: transcranial magnetic stimulation
% Δ: percent change from baseline
Δ_B-A_: net difference between visits
|Δ_B-A_|: absolute difference between visits
